# Autonomic Nervous System Maturation and Emotional Coordination in Interactions of Preterm and Full-Term Infants With Their Parents: Protocol for a Multimethod Study

**DOI:** 10.2196/28089

**Published:** 2021-04-12

**Authors:** Christina Koumarela, Theano Kokkinaki, Giorgos Giannakakis, Katerina Koutra, Eleftheria Hatzidaki

**Affiliations:** 1 Laboratory of Applied Psychology Department of Psychology University of Crete Rethymnon Greece; 2 Institute of Computer Science Foundation of Research and Technology Heraklion Greece; 3 Department of Psychology University of Crete Rethymnon Greece; 4 Department of Neonatology, Neonatal Intensive Care Unit School of Medicine University of Crete Heraklion Greece

**Keywords:** preterm infants, heart rate variability, emotional coordination, developmental outcomes

## Abstract

**Background:**

There is limited knowledge on the physiological and behavioral pathways that may affect the developmental outcomes of preterm infants and particularly on the link between autonomic nervous system maturation and early social human behavior. Thus, this study attempts to investigate the way heart rate variability (HRV) parameters are related to emotional coordination in interactions of preterm and full-term infants with their parents in the first year of life and the possible correlation with the developmental outcomes of infants at 18 months.

**Objective:**

The first objective is to investigate the relationship between emotional coordination and HRV in dyadic full-term infant–parent (group 1) and preterm infant–parent (group 2) interactions during the first postpartum year. The second objective is to examine the relationship of emotional coordination and HRV in groups 1 and 2 in the first postpartum year with the developmental outcomes of infants at 18 months. The third objective is to investigate the effect of maternal and paternal postnatal depression on the relation between emotional coordination and HRV in the two groups and on developmental outcomes at 18 months. The fourth objective is to examine the effect of family cohesion and coping on the relation between emotional coordination and HRV in the two groups and on developmental outcomes at 18 months.

**Methods:**

This is an observational, naturalistic, and longitudinal study applying a mixed method design that includes the following: (1) video recordings of mother-infant and father-infant interactions at the hospital, in the neonatal period, and at home at 2, 4, 6, 9, and 12 months of the infants’ life; (2) self-report questionnaires of parents on depressive symptoms, family cohesion, and dyadic coping of stress; (3) infants’ HRV parameters in the neonatal period and at each of the above age points during and after infant-parent video recordings; and (4) assessment of toddlers’ social and cognitive development at 18 months through an observational instrument.

**Results:**

The study protocol has been approved by the Research Ethics Committee of the University of Crete (number/date: 170/September 18, 2020). This work is supported by the Special Account for Research Funds of the University of Crete (grant number: 10792-668/08.02.2021). All mothers (with their partners) of full-term and preterm infants who give birth between March 2021 and January 2022 at the General University Hospital of Crete (northern Crete, Greece) will be invited to participate. The researcher will invite the parents of infants to participate in the study 1 to 2 days after birth. Data collection is expected to be completed by March 2023, and the first results will be published by the end of 2023.

**Conclusions:**

Investigating the regulatory role of HRV and social reciprocity in preterm infants may have implications for both medicine and psychology.

**International Registered Report Identifier (IRRID):**

PRR1-10.2196/28089

## Introduction

Preterm infants are at increased risk of a range of developmental outcomes at neurological, cognitive, social competence, socioemotional, and behavioral levels [1-3]. Both behavioral and physiological pathways may be connected with the adverse developmental outcomes of preterm infants. At the behavioral level, preterm birth disrupts the emergence of parent-infant emotional synchrony [4-8]. At the physiological level, the early disruption of autonomic nervous system (ANS) development limits its capacity to respond to the environment [2]. Heartrate variability (HRV), that is, the distribution of the time interval between successive heartbeats (R-R intervals), constitutes a measure of ANS functional maturation and sympathetic nervous system (SNS)/parasympathetic nervous system (PNS) interplay, and is an index of the PNS [2,9,10]. HRV is under the control of the PNS branch of the ANS. The vagus nerve is the main nerve of the PNS. Parasympathetic activity is referred to as vagal tone and shows the contribution of the vagus nerve to cardiac functioning [9]. Gestational age in preterm infants greatly correlates with HRV parameters, with a lower gestational age associated with lower HRV [11,12]. Further, the PNS is part of the motor pathway that is connected to facial muscles that support successful interpersonal engagement, such as facial expressions [13]. Thus, higher vagal activity has been connected with better social skills, and cardiac vagal tone monitoring in young infants constitutes an index of their capacity to regulate emotional states via facial expressivity [14,15].

In addition, ex utero third trimester development in a preterm newborn is vulnerable to developmental disruption from a variety of environmental influences [2]. This is important because parents of preterm children experience higher levels of depression and anxiety, and poorer family functioning in infancy and early childhood when compared with parents of full-term children [16,17].

The relation between a young infant’s facial expressions of emotion and autonomic activity has been rarely investigated [4,15,18]. To our knowledge, only two studies have focused on the relationship between vagal activity and *dyadic mother-infant regulatory processes*, and they showed that (1) the cardiac vagal tone of 6-month-old infants was linked to more symmetrical features versus disruptive patterns of communication in mother-infant dyads [19] and (2) newborn vagal activity predicts mother-infant synchrony at 3 months [20].

Infant ANS maturation along with maternal mental health (depression and anxiety) seem to intervene in the relationship between HRV/vagal activity and infant or maternal emotional expressions. In a previous study [4], the vagal tone of preterm infants was shown to be lower than that of full-term infants. Maternal behavior (gaze, affect, touch, and talk) was more frequent among preterm infants with high vagal tone than those with low vagal tone, while preterm infants with low vagal tone received the lowest amount of maternal behavior at 3 months. In the same group, infant vagal tone along with maternal depressive symptoms were predictive of mother-infant gaze synchrony.

Newborns of mothers with high levels of depressive symptoms and depressed mothers had lower vagal tone than newborns of nondepressed mothers [21,22]. Further, in the course of interactions of 3-month-old infants with their nondepressed and low-depressed mothers, infant vagal tone was associated with positive affective states. Infants with greater vagal activity expressed more positive affective states. Relevant correlations were not found in the depressed group, which implies decoupling between vagal tone and facial expressivity in infants of depressed mothers [23]. Lower vagal tone was evidenced in 6-month-old infants of depressed mothers compared with nondepressed mothers, but it was not noted at 3 months. Higher vagal tone at 6 months was related to more positive vocalizations. Though a large increase in baseline vagal activity occurred from 3 to 6 months in infants of nondepressed mothers, this increase did not occur in infants of depressed mothers [24]. Further, newborns of high anxiety mothers (compared with low anxiety mothers) had lower vagal tone [25].

To our knowledge, fathers were included in only two relevant studies. First-time fathers’ heart rates increased during interactions with their newborn infants in a controlled hospital setting. Changes in fathers’ cardiovascular physiology were not related to paternal smiling [26]. Further, neonatal vagal tone predicted gaze synchrony in interactions of 3-month-old full-term and preterm infants with their mothers and fathers, despite its marginal contribution to father-infant synchrony in full-term infants. Maternal, but not paternal, postnatal depression predicted parent-infant gaze synchrony at 3 months [4].

A limited number of studies has investigated the association between interparental functioning with infant emotional and physiological regulation. Six-month-old infants in families with higher marital conflict showed lower baseline vagal tone and poorer emotional regulation [27]. Further, 6-month-old infants coming from families with higher levels of parent conflict showed lower mean levels of baseline respiratory sinus arrhythmia (RSA, ie, heart rate variation according to inspiration [acceleration] and expiration [slowing down] [2,9]), a lesser degree of RSA withdrawal in response to mothers’ still face, and a lesser degree of RSA activation in contexts in which infants were interacting with mothers. No relevant effects were found among parent conflict, infants’ behavior, mothers’ positive affect, and dyadic synchrony [28].

The proposed research, which is the first of its kind in Greece, is interdisciplinary between psychology and medicine in the basic idea, in theoretical frameworks that drive it, in the methodology, and in the implications of the results. In particular, the proposed research is interdisciplinary in the basic idea because it focuses on both expressive behaviors (facial expressions of emotion and emotional coordination) and physiological indexes of ANS (HRV). In addition, the features of preterm infants invite the psychology-medicine collaboration because (1) they possess behavioral characteristics and have neurological immaturity that may contribute in making them difficult interactive partners [29] and increase their health risks [11] and (2) there is a steady increase in neonatal survival rates as the advent of modern intensive care for preterm infants is related to a growing concern for preterm infants’ developmental outcomes and quality of life [1]. Further, this research is interdisciplinary in theoretical frameworks because it is conceived in accordance with the psychobiological theory of innate intersubjectivity developed by Trevarthen et al [30-32] and the biobehavioral polyvagal theory developed by Porges et al [13,33,34]. The psychobiological theory of innate intersubjectivity posits that infants possess innate motives expressed with qualities of emotion and adapted to perceive, respond to, attract, and influence how other persons feel and what they, in response, will perceive and do. Sensitivity for regulation of both subjective and intersubjective impulses and feelings undergoes age-related changes attributable to developments in the body and in the motivating processes of the brain. In connection to this, the polyvagal theory links the maturational shifts in neural regulations of the ANS with infant self-regulatory and social engagement skills [13]. In addition, this study is interdisciplinary in the methodology. In particular, this is a multimethod, observational, naturalistic, and longitudinal study. The methodological strategy will include (1) video recordings of the dyadic mother-infant and father-infant face-to-face interaction in the neonatal period (at hospital) and at 2, 4, 6, 9, and 12 months (at the family’s home); (2) physiological measures, that is, HRV of infants at the neonatal period and at each of the above age points; (3) one observational instrument in order to assess the toddler’s social and cognitive development at 18 months; and (4) self-report questionnaires on postnatal depression, family cohesion, and coping between partners. Finally, investigating the regulatory role of HRV/social reciprocity in preterm infants may have implications for both medicine and psychology. In particular, different neonatal pathological states have been associated with a reduction in HRV in preterm infants, and an improvement in health conditions is followed by changes in HRV, which can be used as a possible prognostic marker [11]. Further, the findings of this study may extend our understanding on the early onset of neuropsychiatric disorders given that this may be related to factors that alter ANS maturation and limbic system functions. In addition, the findings of this study may inform interventions to promote the development of preterm infants given that children at increased risk for neuropsychiatric disorders are those with a history of prematurity [2].

We will use detailed measures of timing to analyze the way emotional coordination in spontaneous interactions of preterm and full-term infants with their parents at home in Greece (across the first year of life) is related to HRV parameters and the possible correlation with the developmental outcomes of infants at 18 months. This study extends beyond the current state-of-the-art knowledge in the following ways:

1. The inclusion of microanalysis of face-to-face father-preterm infant interaction may help in the understanding of the psychological aspects of fathers of preterm infants given that fathers face different emotional experiences than mothers after preterm birth [35,36].
2. When young infants are observed responding to sensitive caregivers with mutual concern in a familiar environment, the infants show active emotional initiative with a great variety of facial expressions of emotions [37], and they have a different motivation as compared with that in a laboratory setting [38].
3. The longitudinal design of this study is important given that regulation of intersubjective feelings undergoes age-related changes attributable to developments in the body and in the motivating processes of the brain in the first postpartum year [31].

In the absence of relevant studies in Greece and given that certain cultural elements may have the potential to promote preterm infants’ development, investigating the factors that affect the development of preterm infants is important because preterm births constitute a major public health issue [39].The main aim of this study is to investigate the way HRV parameters are related to emotional coordination in interactions of preterm and full-term infants with their parents in the first year of life and the possible correlation with the developmental outcomes of infants at 18 months.

## Methods

### Selection Criteria

#### Inclusion Criteria

Full-term infants (group 1) are eligible to be included in this study if they have no medical complications. Preterm infants (group 2) are eligible to be included in this study if they meet the criterion of gestational age (32-37 weeks). We will focus on preterm infants, rather than extremely preterm infants, given variations between the two groups on communicative abilities and perceived social support of mothers [40,41].

Mothers and fathers are eligible to be included in this study if they meet the following criteria: (1) both are born and have grown up in Greece; (2) they are married to each other; (3) both have given their consent to participate in the study; (4) at least one parent is employed; and (5) both are older than 20 years of age. Greek nationality of participant parents is important given the focus of this study on the way cultural elements of Greece may intervene in infant development.

#### Exclusion Criteria

Infants will be excluded from the study if (1) they have perinatal asphyxia; (2) they have neurological pathologies; (3) they experience malformation syndromes and major congenital malformations; (4) they have sensory deficits; (5) they present metabolic genetic disease; (6) they have central nervous system infection; or (7) they have a birth weight less than 2500 g in case of full-term birth [42].

Mothers and fathers will be excluded from the study if (1) they are not of Greek nationality; (2) they have a psychiatric illness; (3) they have issues with drug or substance abuse; (4) they are not living together; (5) they are not biological parents; and (6) they are a same-sex couple. Participation in this study will presuppose that both infants and parents will meet the inclusion and exclusion criteria.

### Recruitment

All mothers (with their partners) of full-term and preterm infants who will give birth between March 2021 and January 2022 at the General University Hospital of Crete (northern Crete, Greece) will be invited to participate in the study. The researcher will invite the parents of infants to participate in the study 1-2 days after birth.

A minimum of 50 mothers, 50 fathers, and 50 infants (N=150) will participate in the study in two groups. Group 1 will include 25 parents and their infants born at full term (≥37 weeks gestational age) with no medical complications. Group 2 will include 25 parents and their preterm infants (32-37 weeks gestational age). The two groups will be matched for demographic variables and infant gender. This sample size is in line with previous relevant studies in Greece and in other countries, and it is adequate to perform parametric statistical tests [35,43].

### Demographic and Socioeconomic Characteristics of the Sample

Before hospital discharge, data will be collected from the mothers on the parents’ demographic and socioeconomic characteristics, mothers’ obstetric history, and infants’ characteristics (gestational age, weight, height, head circumference, and Apgar score at 1 and 5 minutes). In the case of preterm infants, information will also include the duration and the conditions of infant hospitalization (in case of hospitalization).

### Objectives, Procedure, and Measures

The first objective is to compare the relationship between emotional coordination and HRV in dyadic full-term infant–parent (group 1) and preterm infant–parent (group 2) interactions in the course of the first postpartum year.

For the first objective of this study, each full-term neonate/infant–parent dyad will be video recorded at six age points, that is, at the neonatal period (in the hospital) and at five important age-related transitions in the development of communication in the first postpartum year [31] (2, 4, 6, 9, and 12 months [chronological age]) at home. For preterm neonates/infants, video recordings will be carried out around 36 weeks postmenstrual age at the hospital and at 2, 4, 6, 9, and 12 months (corrected age) at home [44].

In particular, before discharge, at the hospital, each neonate-parent dyad will be video recorded for a 5-minute period. Before, during, and after each infant-parent video recording, the neonate’s HRV recording will be obtained by measuring short-term variability, which provides important information about the maturation of the ANS in newborns [11]. Before the video recording, the HRV evaluation will be performed under resting conditions in the supine position for 5 minutes, and infants will be in this position at least 5 minutes before the measurement (gold standard [9]). Similarly, after the end of the video recording, we will evaluate HRV parameters for 5 minutes. Whenever possible, we will control for the time of the day of the assessment, and we will follow consistent procedures for all participants [9].

A validated portable electrocardiography (ECG) device will be used to collect ECG recordings. ECG is considered the gold standard providing accurate measurements to perform the appropriate HRV analysis offline [45].

The order of mother-infant and father-infant interactions will be counterbalanced with a 5-minute rest for the neonates between the two video recordings. Parents will be told “Play with your baby.” Video recordings at the neonatal period will be scheduled according to mothers’ and fathers’ availability and the infants’ alertness. Parent-neonate free interactions will be carried out on the third postpartum day for the full-term group and the day prior to discharge for the preterm group.

For video recordings at home, parents will be told “Play as you normally do with your baby.” The recording will take place in a room and in a position chosen by the parent, prohibiting any third-party intervention. Given that infant state and age constitute factors that influence arousal, attention, and affect [46], we will vary the duration of video recordings across the age range of this study. Thus, for younger infants aged 2 and 4 months, each video recording will last 8 minutes in order to respect their early signs of agitation after brief face-to-face exchanges with their parents [32]. In the meanwhile, we will video record interactions of parents with older infants (aged 6, 9, and 12 months) for 10 minutes in order to predict periods of infant exploration of surroundings, which intensify after the middle of the Period of Games I (3-6 months, characterized by a transformation of the infant’s motivation, which results in both increasing exploration of the environment and effective manipulation of objects) [47], and to ensure an 8-minute face-to-face interaction. The total duration for parent-infant interactions will be 4600 minutes of video data, equally distributed between periods with the mother and father and between the two groups. HRV will be evaluated following the same procedure as in the neonatal period.

The second objective is to examine the relationship of emotional coordination and HRV in groups 1 and 2 in the first postpartum year with the developmental outcomes of infants at 18 months. At 18 months, the social and cognitive development of toddlers will be assessed at home through the administration of Bayley Scales of Infant and Toddler Development, 3rd edition [48].

The third objective is to investigate the effect of maternal and paternal postnatal depression on the relation between emotional coordination and HRV in the two groups and on developmental outcomes at 18 months.

According to a relevant literature review (mentioned in the Introduction), limited information on the relation between mood disorders and HRV is focused mainly on depression rather than on other mood disorders (eg, anxiety). Considering this, parents will be asked to complete (1) the Edinburgh Postnatal Depression Scale (EPDS [49]) adapted for the Greek population [50,51] (at 2 months), which is an instrument that screens women/men for postnatal depression, and (2) the Beck Depression Inventory-II (BDI-II [52]) adapted for the Greek population [53] (at 18 months), which is a self-report scale assessing symptoms of depression.

The fourth objective is to examine the effect of family cohesion and coping on the relation between emotional coordination and HRV in the two groups and on developmental outcomes at 18 months. At 2 and 18 months, parents will be asked to complete (1) the Greek version of the Family Adaptability and Cohesion Evaluation Scale (FACES-IV Package) [54-56] to measure family functioning in terms of cohesion and flexibility and (2) the Greek version of the Dyadic Coping Inventory (DCI) [57,58] for the assessment of dyadic stress coping among the couple.

### Data Analysis

#### Video Coding

Coding of parent-newborn interactions will be carried out using the Mother-Newborn Coding System of the Coding Interactive Behavior (CIB) Manual developed by Feldman in 1998 [59]. For each 30-second period, four maternal/paternal categories and one newborn category will be coded. Categories for the mother/father include gaze, affect, talk, and touch. Infant state is coded as fussy, cry, drowsy, sleep, alert to mother/father, or alert to the environment. Maternal/paternal affiliative behavior will include the sum proportions of maternal/paternal gaze at the infant’s face, positive affect, vocalization, and affectionate touch. Emotional/social coordination will be examined according to conditional probabilities that come from the time of maternal/paternal affiliative behavior during moments when the newborn is in an alert state to the mother/father or to the environment, and the time the mother/father expresses affiliative behavior when the infant is in another state.

Microanalysis of infant and parental facial expressions of emotions at 2, 4, and 6 months will be carried out according to a coding system that has been constructed by ΤΚ [60-62]. This system adapted and extended certain parts of the categorization system developed by previous authors [63-65]. In particular, microanalysis and coding of infant-parent emotional coordination at 2, 4, and 6 months will be carried out as follows. First, parental infant-directed speech and infant (vocalizations/nonspeech) sounds will be transcribed from the video recording by the researcher. Thereafter, they will be checked for accuracy by an assistant. Second, written verbatim accounts of parental infant-directed speech will be classified into “content categories” and then into “thematic sequences” and “focus categories” (to an accuracy of 1/25th of a second). Third, each focus category and within thematic sequence will be segmented into units and subunits of analysis, according to the duration of the pause preceding and following each thematic sequence. If the pause between successive subunits/thematic sequences is shorter than or equal to 2 seconds, these will be grouped within one unit. Fourth, within each subunit of analysis, infant and maternal/paternal facial expressions of emotions will be coded according to the type of facial expressions of emotions, the shifts in the frequency of facial expressions of emotions, and the direction of intensity change. Thereafter, interpersonal engagement categories according to the types of facial expressions of emotions (happy, interest, neutral, and sad or withdrawn) will be coded as *matching*, *completion*, and *nonmatching*. Interpersonal engagement categories according to the shifts in the frequency of facial expressions of emotions will be coded as *synchrony*. Interpersonal engagement categories according to the direction of intensity change will be coded as *attunement*. In *matching*, one partner expresses the type of facial expression of the emotion of the other partner. In *completion*, one partner expresses a positive valence of facial expression of emotion (“pleasure” or “interest”) in immediate response to the other. In *synchrony*, the two partners match the timing of change of emotional expressions with each other. In *attunement*, one partner expresses the shifts in the direction of the emotional intensity of the other partner. Emotional coordination is signified by these aspects. Instances in which the two partners do not match the type of facial expression of emotion or do not synchronize their shifts of emotions, or one partner is not attune to the direction of the emotional intensity of the other partner will be termed as *nonmatching*. Each emotional coordination category will be indexed by two conditional probabilities, for example, for emotional matching, the infant expresses pleasure given that the mother expresses pleasure and the infant expresses pleasure given that the father expresses the same facial expression of emotion (pleasure). These probabilities provide evidence of the co-occurrence of the same facial expression of emotion between the two partners and show the proportion of time out of the entire interaction that both the parent and the infant match their emotional expressions. For microanalysis of parent-infant emotional coordination at 9 and 12 months, all necessary adjustments will be made to the above coding scheme according to changes in the infant’s motivation for communication and exploration of the environment after 6 months.

Concurrent with the microanalysis of infant-parent emotional coordination, HRV parameters will be measured according to the analysis described in the subsection “HRV Analysis” below.

#### Interobserver Reliability

To measure interobserver reliability (Cohen κ), a second observer who will be trained in the use of the coding system, but will not be aware of the hypotheses under investigation, will score a random sample of 33% of the video files. Interobserver reliability will be assessed separately for the types of facial expressions, the frequency of shifts of emotions, and the categories of emotional intensity.

### Quantitative Data Analysis

#### HRV Analysis

Heart activity will be recorded concurrently with video recordings (ie, 5 minutes, 8 minutes, and 10 minutes) with a sampling frequency of 256 Hz. The recording length is selected as it has been proven that normally HRV parameters need at least 5 minutes in order to have sufficient statistical power [66]. However, enhanced spectral resolution using autoregressive analysis will be performed. Thus, sliding temporal windows of Δ*t*=30 s will be investigated in order to track HRV temporal dynamics. This temporal window length is sufficient for the temporal evolution of HRV as referred in relevant studies [67,68].

Artifacts (mainly due to body motion) will be automatically suppressed. Time series with less than 10% of artifacts will be included for analysis [69]. In addition, ectopic beats will be identified and will be excluded from the analysis [70]. The HRV parameters in the time domain and the frequency domain, as well as nonlinear indices will be analyzed [9,45,66]. In connection with the focus of this study on infant vagal tone, certain relevant HRV indices will be of interest for analysis. Thus, for the time domain, we will analyze mean heart rate, SDNN (standard deviation of all RR intervals), rMSSD (root mean square of successive differences), and pNN50 (percentage of adjacent RR intervals with a duration difference greater than 50 ms). The rMSSD and pNN50 indices reflect vagal tone, and SDNN reflects the SNS and PNS [9,69].

For the frequency domain, low frequency (LF, 0.04-0.15 Hz), high frequency (HF, 0.15-0.40 Hz), and the LF/HF ratio will be analyzed. HF reflects vagal tone, while LF and the LF/HF ratio reflect a mix of sympathetic and vagal activity [9,69]. Autoregressive analysis will be preferred as it provides enhanced spectral resolution compared with Fast Fourier Transform–based spectrum analysis for the frequency domain of HRV parameters [9,67,71]. Given that infants breathe faster, bands will be adjusted accordingly, and thus, we will move the boundaries of the bands to 0.24-1.04 Hz at rest [72].

For the nonlinear HRV analysis, the following parameters derived from the Poincaré plots, which are assumed to be indicators of vagal activity [9], will be estimated: SD1 (reflecting the PNS), SD2 (reflecting the SNS), and SD1/SD2 (the relationship of the balance between the PNS and SNS) [69]. Nonlinear indices are complementary HRV indicators to enhance the estimation of the participants’ physiological status [9].

### Statistical Analysis

The first objective of this study is to investigate differences in emotional coordination and HRV parameters between the two groups of infants (normal and preterm) over the first year of life. For the assessment of preterm infants’ social interaction capability, mother/father-infant dyads will be analyzed in order to evaluate the “emotional coordination” and “nonmatching” of facial expressions of emotions during spontaneous interactions [62]. First, temporal statistical analysis (analysis of variance [ANOVA]) will be used to evaluate parent-infant synchrony (through HRV/emotional coordination measures) for each group of infants across the first year [20,73]. Second, trend analysis for HRV measurements at 2, 4, 6, 9, and 12 months will be performed in order to observe patterns of developmental changes from the first month to the end of the first year. Third, correlation analysis will be conducted to examine the intercorrelations between the variables of the study [20,74]. Lastly, we will explore whether synchrony of the interaction is more prevalent among the group of infants demonstrating higher HRV on dividing the data into high and low HRV groups [20,74].

The second objective is the investigation of possible correlations between emotional coordination/HRV and developmental outcomes at 18 months of age. A correlation analysis will be used to measure potential linear correlation between emotional coordination/HRV and developmental outcomes [75]. In addition, two-way ANOVA and multivariate analysis of variance (MANOVA) will be utilized to examine the potential effect of early mother/father-infant interactive patterns and HRV measures on infant developmental outcomes at 18 months of age [1,74].

The third objective focuses on the examination of the mediating role of parental postpartum depression and family functioning/stress coping in relation to emotional coordination/HRV and developmental outcomes at 18 months. The Wilcoxon test and bivariate correlations will be used to detect potential different patterns of the study variables between normal and high-risk groups under this condition [4,28,76,77]. Analysis of covariance (ANCOVA) will be used to compute differences in the study variables between the term and preterm infant groups [4,78]. To examine the moderating effect of parental postpartum depressive symptoms, family functioning, and stress coping on emotional coordination/HRV and the developmental outcomes of toddlers, a hierarchical linear regression analysis will be performed [4,20].

## Results

**Figure 1:**
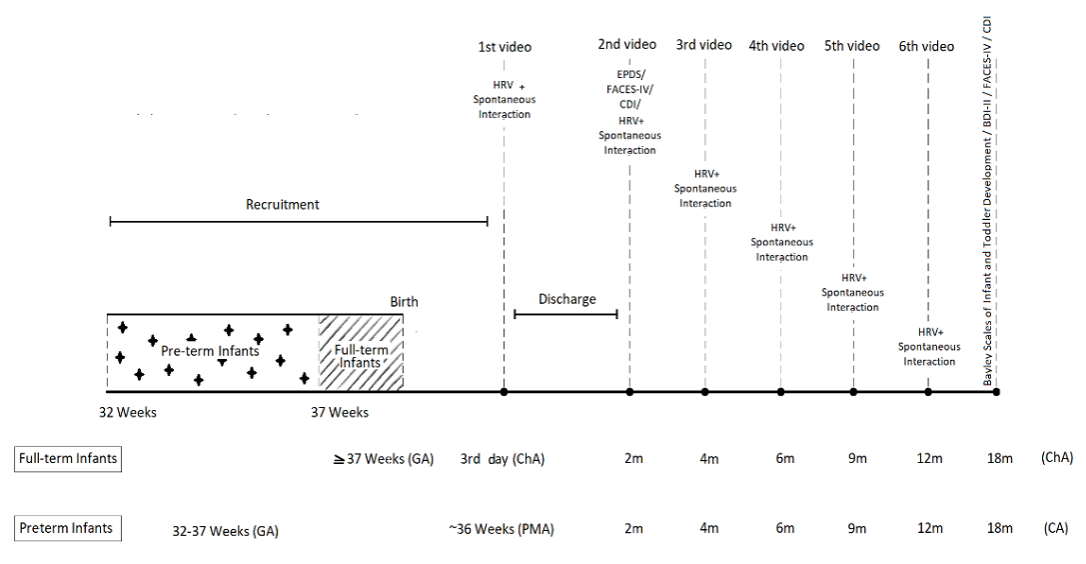
Timeline of the study procedure. BDI: Beck Depression Inventory; CA: Corrected Age; DCI: Dyadic Coping Inventory; ChA: Chronological Age; EPDS: Edinburgh Postnatal Depression Scale; FACES: Family Adaptability and Cohesion Evaluation Scale; GA: Gestational Age; HRV: Heart Rate Variability; PMA: Postmenstrual Age.

The timeline of the study procedure is depicted in Figure 1.

The study protocol has been approved by the Research Ethics Committee of the University of Crete (number and date of decision: 170/September 18, 2020). This work is funded by the Special Account for Research Funds of the University of Crete (grant number: 10792-668/08.02.2021).

All mothers (with their partners) of full-term and preterm infants who will give birth between March 2021 and January 2022 at the General University Hospital of Crete (northern Crete, Greece) will be invited to participate in the study. Data collection is expected to be completed by March 2023, and the first results will be published by the end of 2023.

## Discussion

There is evidence that higher vagal activity is connected with better social skills, and cardiac vagal tone monitoring for young infants constitutes an index of their capacity to regulate emotional states via facial expressivity [14,15]. In the meanwhile, a limited number of studies show that infant ANS maturation along with maternal/paternal mental health (depression) and interparental functioning seem to intervene in the relationship between HRV/vagal activity and infant or maternal/paternal emotional expressions [4,21-24,27]. This may have long-term implications for infant cognitive and socioemotional development. In order to fill this gap, the main aim of this study is to investigate the way HRV parameters are related to emotional coordination in interactions of preterm and full-term infants with their parents in the first year of life and the possible correlation with developmental outcomes at 18 months. Toward this direction, our study has four objectives. The first objective is to investigate the relationship between emotional coordination and HRV in dyadic full-term infant–parent (group 1) and preterm infant–parent (group 2) interactions in the course of the first postpartum year. The second objective is to examine the relationship of emotional coordination and HRV in groups 1 and 2 in the first postpartum year with the developmental outcomes of infants at 18 months. The third objective is to investigate the effect of maternal and paternal postnatal depression on the relation between emotional coordination and HRV in the two groups and on developmental outcomes at 18 months. The fourth objective is to examine the effect of family cohesion and coping on the relation between emotional coordination and HRV in the two groups and on developmental outcomes at 18 months.

The proposed research is interdisciplinary in the implications of the results. In particular, investigating the regulatory role of HRV and social reciprocity in preterm infants may have implications for both medicine and psychology. With regard to medicine, different neonatal pathological states have been associated with a reduction in HRV in preterm infants, and an improvement in health conditions is followed by changes in HRV, which can be used as a possible prognostic marker. With regard to psychology, the findings of this study may extend our understanding on the early onset of neuropsychiatric disorders given that this may be related to factors that alter ANS maturation and limbic system functions. Further, the results of this study may contribute to the development of neonatal telemedicine services with the aim to educate new parents and to improve the care of preterm and full-term neonates [79].

## Abbreviations

ANCOVA: analysis of covariance
ANOVA: analysis of variance
ANS: autonomic nervous system
BDI: Beck Depression Inventory
CIB: Coding Interactive Behavior
DCI: Dyadic Coping Inventory
ECG: electrocardiography
EPDS: Edinburgh Postnatal Depression Scale
FACES: Family Adaptability and Cohesion Evaluation Scale
HF: high frequency
HRV: heart rate variability
LF: low frequency
MANOVA: Multivariate Analysis of Variance
PNN50: percentage of adjacent RR intervals with a duration difference greater than 50 ms
PNS: parasympathetic nervous system
rMSSD: root mean square of successive differences
RSA: respiratory sinus arrhythmia
SDNN: standard deviation of RR intervals
SNS: sympathetic nervous system

## References

[1] Forcada-Guex M, Pierrehumbert B, Borghini A, Moessinger A, Muller-Nix C (2006). Early dyadic patterns of mother-infant interactions and outcomes of prematurity at 18 months. Pediatrics.

[2] Mulkey SB, du Plessis AJ (2019). Autonomic nervous system development and its impact on neuropsychiatric outcome. Pediatr Res.

[3] Pierrehumbert B, Nicole A, Muller-Nix C, Forcada-Guex M, Ansermet F (2003). Parental post-traumatic reactions after premature birth: implications for sleeping and eating problems in the infant. Arch Dis Child Fetal Neonatal Ed.

[4] Feldman R, Eidelman AI (2007). Maternal postpartum behavior and the emergence of infant-mother and infant-father synchrony in preterm and full-term infants: the role of neonatal vagal tone. Dev Psychobiol.

[5] Gerner EM (2001). Emotional interaction in a group of preterm infants at 3 and 6 months of corrected age. Inf. Child Develop.

[6] Malatesta CZ, Grigoryev P, Lamb C, Albin M, Culver C (1986). Emotion Socialization and Expressive Development in Preterm and Full-Term Infants. Child Development.

[7] Sansavini A, Zavagli V, Guarini A, Savini S, Alessandroni R, Faldella G (2015). Dyadic co-regulation, affective intensity and infant's development at 12 months: A comparison among extremely preterm and full-term dyads. Infant Behav Dev.

[8] Stefana A, Lavelli M (2018). What is hindering research on psychological aspects of fathers of premature infants?. Minerva Pediatr.

[9] Laborde S, Mosley E, Thayer JF (2017). Heart Rate Variability and Cardiac Vagal Tone in Psychophysiological Research - Recommendations for Experiment Planning, Data Analysis, and Data Reporting. Front Psychol.

[10] Giannakakis G, Grigoriadis D, Giannakaki K, Simantiraki O, Roniotis A, Tsiknakis M (2019). Review on psychological stress detection using biosignals. IEEE Trans. Affective Comput.

[11] Javorka K, Lehotska Z, Kozar M, Uhrikova Z, Kolarovszki B, Javorka M, Zibolen M (2017). Heart rate variability in newborns. Physiol Res.

[12] Suga A, Uraguchi M, Tange A, Ishikawa H, Ohira H (2019). Cardiac interaction between mother and infant: enhancement of heart rate variability. Sci Rep.

[13] Porges SW, Furman SA (2011). The Early Development of the Autonomic Nervous System Provides a Neural Platform for Social Behavior: A Polyvagal Perspective. Infant Child Dev.

[14] Field T, Diego M (2008). Vagal activity, early growth and emotional development. Infant Behav Dev.

[15] Stifter CA, Fox NA, Porges SW (1989). Facial expressivity and vagal tone in 5- and 10-month-old infants. Infant Behavior and Development.

[16] Feldman R (2007). Maternal versus child risk and the development of parent–child and family relationships in five high-risk populations. Develop. Psychopathol.

[17] Treyvaud K, Lee KJ, Doyle LW, Anderson PJ (2014). Very preterm birth influences parental mental health and family outcomes seven years after birth. J Pediatr.

[18] Field T, Woodson R, Greenberg R, Cohen D (1982). Discrimination and imitation of facial expression by neonates. Science.

[19] Porter CL (2003). Coregulation in mother-infant dyads: links to infants' cardiac vagal tone. Psychol Rep.

[20] Feldman R (2006). From biological rhythms to social rhythms: Physiological precursors of mother-infant synchrony. Dev Psychol.

[21] Field T, Diego M, Dieter J, Hernandez-Reif M, Schanberg S, Kuhn C, Yando R, Bendell D (2004). Prenatal depression effects on the fetus and the newborn. Infant Behavior and Development.

[22] Aaron Jones N, Field T, Fox NA, Davalos M, Lundy B, Hart S (1998). Newborns of mothers with depressive symptoms are physiologically less developed. Infant Behavior and Development.

[23] Pickens JN, Field T (1995). Facial expressions and vagal tone of infants of depressed and non-depressed mothers. Early Dev. Parent.

[24] Field T, Pickens J, Fox NA, Nawrocki T, Gonzalez J (2009). Vagal tone in infants of depressed mothers. Dev Psychopathol.

[25] Field T, Diego M, Hernandez-Reif M, Schanberg S, Kuhn C, Yando R, Bendell D (2003). Pregnancy anxiety and comorbid depression and anger: effects on the fetus and neonate. Depress Anxiety.

[26] Jones LC, Thomas SA (1989). New fathers' blood pressure and heart rate: relationships to interaction with their newborn infants. Nurs Res.

[27] Porter C, Wouden-Miller M, Silva S, Porter A (2003). Marital harmony and conflict: Links to infants' emotional regulation and cardiac vagal tone. Infancy.

[28] Moore GA (2010). Parent conflict predicts infants' vagal regulation in social interaction. Dev Psychopathol.

[29] Singer LT, Fulton S, Davillier M, Koshy D, Salvator A, Baley JE (2003). Effects of infant risk status and maternal psychological distress on maternal-infant interactions during the first year of life. J Dev Behav Pediatr.

[30] Trevarthen C, Aitken K, Vandekerckhove M, Delafield-Butt J, Nagy E, Cicchetti D, Cohen DJ (2015). Collaborative Regulations of Vitality in Early Childhood: Stress in Intimate Relationships and Postnatal Psychopathology. Developmental Psychopathology: Volume Two: Developmental Neuroscience, Second Edition.

[31] Trevarthen C, Aitken K, Heimann M, Plooij FX (2003). Regulation of Brain Development and Age-Related Changes in Infants' Motives: The Developmental Function of Regressive Periods. Regression Periods in Human infancy.

[32] Trevarthen C, Scherer K, Ekman P (1984). Emotions in infancy: Regulators of contact and relationships with persons. Approaches to Emotion.

[33] Porges S (2003). Social engagement and attachment: a phylogenetic perspective. Ann N Y Acad Sci.

[34] Porges SW (2009). The polyvagal theory: new insights into adaptive reactions of the autonomic nervous system. Cleve Clin J Med.

[35] Stefana A, Lavelli M (2017). Parental engagement and early interactions with preterm infants during the stay in the neonatal intensive care unit: protocol of a mixed-method and longitudinal study. BMJ Open.

[36] Stefana A, Padovani EM, Biban P, Lavelli M (2018). Fathers' experiences with their preterm babies admitted to neonatal intensive care unit: A multi-method study. J Adv Nurs.

[37] Trevarthen C, Kokkinaki T, Fiamenghi G, Nadel J, Butterworth G (1999). What infants' imitations communicate: With mothers, with fathers and with peers. Cambridge studies in cognitive perceptual development. Imitation in infancy.

[38] Beebe B, Jaffe J, Feldstein S, Mays K, Alson D, Field T, Fox N (1984). Interpersonal timing: The application of an adult dialogue model to mother-infant vocal and kinesic interactions. Infant social perception.

[39] Vlachadis N, Kornarou E, Ktenas E (2013). The preterm births epidemic in Greece. Acta Obstet Gynecol Scand.

[40] Gatta M, Miscioscia M, Svanellini L, Peraro C, Simonelli A (2017). A Psychological Perspective on Preterm Children: The Influence of Contextual Factors on Quality of Family Interactions. Biomed Res Int.

[41] Ribeiro C, Lamônica D (2014). Communicative abilities in premature and extreme premature infants. Rev. CEFAC.

[42] Hughes MM, Black RE, Katz J (2017). 2500-g Low Birth Weight Cutoff: History and Implications for Future Research and Policy. Matern Child Health J.

[43] Kokkinaki T (1998). Emotion and imitation in early infant-parent interaction: a longitudinal and cross-cultural study. Edinburgh Research Archive.

[44] Engle WA, American Academy of Pediatrics Committee on FetusNewborn (2004). Age terminology during the perinatal period. Pediatrics.

[45] de Souza Filho LFM, de Oliveira JCM, Ribeiro MKA, Moura MC, Fernandes ND, de Sousa RD, Pedrino GR, Rebelo ACS (2019). Evaluation of the autonomic nervous system by analysis of heart rate variability in the preterm infants. BMC Cardiovasc Disord.

[46] Field T (1981). Infant arousal attention and affect during early interactions. Advances in Infancy Research.

[47] Trevarthen C (2017). Play with infants: The impulse for human storytelling. The Routledge International Handbook of Early Childhood Play.

[48] Bayley Scales of Infant and Toddler Development. Pearson.

[49] Cox JL, Holden JM, Sagovsky R (1987). Detection of postnatal depression. Development of the 10-item Edinburgh Postnatal Depression Scale. Br J Psychiatry.

[50] Leonardou AA, Zervas YM, Papageorgiou CC, Marks MN, Tsartsara EC, Antsaklis A, Christodoulou GN, Soldatos CR (2009). Validation of the Edinburgh Postnatal Depression Scale and prevalence of postnatal depression at two months postpartum in a sample of Greek mothers. Journal of Reproductive and Infant Psychology.

[51] Vivilaki VG, Dafermos V, Kogevinas M, Bitsios P, Lionis C (2009). The Edinburgh Postnatal Depression Scale: translation and validation for a Greek sample. BMC Public Health.

[52] Beck A, Steer R, Brown G (1996). Manual for the Beck depression inventory-II.

[53] Giannakou M, Roussi P, Kosmides M, Kiosseoglou G, Adamopoulou A, Garyfallos G (2013). Adaptation of the Beck Depression Inventory-II to Greek population. Hellenic Journal of Psychology.

[54] Olson DH (2008). Circumplex Model of Marital and Family Systems. Journal of Family Therapy.

[55] Olson DH, Sprenkle DH, Russell CS (1979). Circumplex model of marital and family system: I. Cohesion and adaptability dimensions, family types, and clinical applications. Fam Process.

[56] Koutra K, Triliva S, Roumeliotaki T, Lionis C, Vgontzas AN (2012). Cross-Cultural Adaptation and Validation of the Greek Version of the Family Adaptability and Cohesion Evaluation Scales IV Package (FACES IV Package). Journal of Family Issues.

[57] Bodenmann G (2008). Dyadic coping and the significance of this concept for prevention and therapy. Zeitschrift für Gesundheitspsychologie.

[58] Roussi P, Karademas E, Falconier MK, Randall AK, Bodenmann G (2016). Dyadic coping in Greek couples. Couples coping with stress: A cross-cultural perspective.

[59] Halevi G, Djalovski A, Kanat-Maymon Y, Yirmiya K, Zagoory-Sharon O, Koren L, Feldman R (2017). The social transmission of risk: Maternal stress physiology, synchronous parenting, and well-being mediate the effects of war exposure on child psychopathology. J Abnorm Psychol.

[60] Kokkinaki T, Vasdekis VGS (2020). Beyond the Words: Comparing Interpersonal Engagement Between Maternal and Paternal Infant-Directed Speech Acts. Front Psychol.

[61] Kokkinaki T, Vasdekis VGS (2014). Comparing emotional coordination in early spontaneous mother–infant and father–infant interactions. European Journal of Developmental Psychology.

[62] Kokkinaki TS, Vasdekis V, Koufaki ZE, Trevarthen CB (2016). Coordination of Emotions in Mother-Infant Dialogues. Inf Child Dev.

[63] Murray L, Field T, Fox N (1985). Emotional regulations of interactions between two-month-oldsand their mothers. Social Perceptions in Infants.

[64] Murray L, Kempton C, Woolgar M, Hooper R (1993). Depressed mothers' speech to their infants and its relation to infant gender and cognitive development. J Child Psychol Psychiatry.

[65] Butler S, McMahon C, Ungerer JA (2003). Maternal speech style with prelinguistic twin infants. Inf. Child Develop.

[66] Malik M, Bigger JT, Camm AJ, Kleiger RE, Malliani A, Moss AJ, Schwartz PJ (1996). Heart rate variability: Standards of measurement, physiological interpretation, and clinical use. European Heart Journal.

[67] Giannakakis G, Tsiknakis M, Vorgia P (2019). Focal epileptic seizures anticipation based on patterns of heart rate variability parameters. Comput Methods Programs Biomed.

[68] Greene BR, de Chazal P, Boylan GB, Connolly S, Reilly RB (2007). Electrocardiogram Based Neonatal Seizure Detection. IEEE Trans. Biomed. Eng.

[69] Selig FA, Tonolli ER, Silva EVC, Godoy MFD (2011). Heart rate variability in preterm and term neonates. Arq Bras Cardiol.

[70] Nabil D, Bereksi Reguig F (2015). Ectopic beats detection and correction methods: A review. Biomedical Signal Processing and Control.

[71] Tarvainen MP, Georgiadis SD, Ranta-Aho PO, Karjalainen PA (2006). Time-varying analysis of heart rate variability signals with a Kalman smoother algorithm. Physiol Meas.

[72] Quintana DS, Alvares GA, Heathers JAJ (2016). Guidelines for Reporting Articles on Psychiatry and Heart rate variability (GRAPH): recommendations to advance research communication. Transl Psychiatry.

[73] Feldman R (2007). Parent-infant synchrony and the construction of shared timing; physiological precursors, developmental outcomes, and risk conditions. J Child Psychol Psychiatry.

[74] Huffman LC, Bryan YE, del Carmen R, Pedersen FA, Doussard‐Roosevelt JA, Forges SW (1998). Infant Temperament and Cardiac Vagal Tone: Assessments at Twelve Weeks of Age. Child Dev.

[75] DiPietro JA, Bornstein MH, Hahn C, Costigan K, Achy-Brou A (2007). Fetal heart rate and variability: stability and prediction to developmental outcomes in early childhood. Child Dev.

[76] Cummings EM, Keller PS, Davies PT (2005). Towards a family process model of maternal and paternal depressive symptoms: exploring multiple relations with child and family functioning. J Child Psychol Psychiatry.

[77] Doussard-Roosevelt JA, McClenny BD, Porges SW (2000). Neonatal cardiac vagal tone and school-age developmental outcome in very low birth weight infants. Dev. Psychobiol.

[78] Kaplan LA, Evans L, Monk C (2008). Effects of mothers' prenatal psychiatric status and postnatal caregiving on infant biobehavioral regulation: can prenatal programming be modified?. Early Hum Dev.

[79] Isetta V, Lopez-Agustina C, Lopez-Bernal E, Amat M, Vila M, Valls C, Navajas D, Farre R (2013). Cost-effectiveness of a new internet-based monitoring tool for neonatal post-discharge home care. J Med Internet Res.

